# Patient outcome scores between 1,2- and 2,3-intercompartmental supra-retinacular artery (ICSRA) pedicled vascularised bone grafts (PVBGs) in the treatment of proximal pole scaphoid fracture non-union – a questionnaire study

**DOI:** 10.1186/s12891-023-06870-4

**Published:** 2023-09-28

**Authors:** Mark Bugeja, Jordan Calleja, Tim Drew, Gor Poghosyan

**Affiliations:** 1 Department of Orthopaedic and Trauma Surgery, University of Dundee, Dundee, DD1 4HN Scotland, UK; 2 Department of Orthopaedic and Trauma Surgery, Mater Dei Hospital, Msida, 2090 MSD Malta

**Keywords:** Scaphoid fracture, Non-union, 1,2-ICSRA, 2,3-ICSRA, Pedicle vascularised bone graft

## Abstract

**Background:**

Numerous studies have been published on the use of 1,2-intercompartmental supra-retinacular artery (ICSRA) as a pedicled vascularised bone graft (PVBG) in scaphoid fracture non-union, however, only very few studies have reported their results of 2,3-ICSRA. The aim of this study was to compare the patient-rated outcome scores between these two PVBGs in proximal pole scaphoid fracture non-union.

**Methods:**

Nineteen patients who underwent surgery for scaphoid non-union between 2017 and 2021 at a single institution were recruited retrospectively in this study. All patients were operated by a one senior orthopaedic surgeon. A mailed questionnaire with the modified mayo wrist (MMW) and the patient rated wrist evaluation (PRWE) scores were sent to the patients.

**Results:**

All patients were males with a mean age of 22.5 years. There was no statistically significant difference in the PRWE score between the two PVBGs. However, a statistically significant difference was found in the MMW score, with the 1,2-ICSRA PVBG having better scores.

**Conclusion:**

Despite the 2,3-ICSRA having a longer arc of rotation, longer nutrient arteries, and is technically easier to incorporate in a PVBG, when compared with the 1,2-ICSRA it did not result in better patient-rated outcome scores.

## Introduction

### Background

The scaphoid is the most common fractured bone in the carpus, accounting for approximately 60–90% of all carpal bone fractures [1–4]. Between 5 and 25% fail to heal, and if left untreated, will progress to wrist osteoarthritis and poor functional outcome [5–10]. In view of this, non-unions should be treated surgically early on. There are several surgical options for scaphoid non-union, however, despite this, the choice of treatment remains controversial.

Pedicled vascularised bone grafts (PVBG) taken from the dorsal distal radius are one of the treatment options for proximal pole scaphoid fracture non-union. Compared to free vascularised bone grafts, PVBG have the advantage of a single surgical incision, no need for vascular anastomosis, and are relatively easy to harvest [8, 11]. The most common PVBG used in proximal pole scaphoid fracture non-union is the 1,2-intercompartmental supra-retinacular artery (ICSRA). This was first discovered in 1991 by Zaidemberg et al. [12] as a small retrograde branch of the radial artery deep in the first extensor compartment at the level of the radial styloid, after using standard latex injection techniques on 20 dissected cadaveric wrists. Zaidemberg et al. [12]were also the first to report the use of this artery as a PVBG, resulting in 100% (11 cases) union and resolution of rest wrist pain. [12].

Sheetz et al. [13] conducted a study on the vascularity of the wrist with the scope of discovering potential PVBG to the carpus. They studied 35 fresh cadaver upper limbs using techniques such as Batson’s compound and latex injections, bone clearing using modified Splateholtz technique, and angiography. They found that the 1,2-ICSRA to be more superficial than reported by Zaidemberg et al. [12] lying on the extensor retinaculum over the tubercle between the first and second extensor wrist compartments. Despite being easy to harvest, its nutrient arteries are very short, the artery itself might not always be present, and its arc of rotation is small. From this study Sheetz et al. [13] discovered the 2,3-ICSRA, a retrograde artery found superficial to the wrist extensor retinaculum over the Lister’s tubercle, between the second and third extensor compartments. Compared to the 1,2-ICSRA, the 2,3-ICSRA has a longer arc of rotation and nutrient arteries, thus theoretically it should result in better outcomes.

Various studies have been published on the use of 1,2-ICSRA in scaphoid fracture non-union, with nearly half of the studies claiming a 100% union rate [14–[26]. However, very few studies have been published on the use of 2,3-ICSRA in scaphoid fracture non-union Tan and Tu [11]. claimed a 92.3% union rate and a 86.5% good to excellent modified mayo wrist (MMW) score with the use of 2,3-ICSRA. [11 While Tu et al. 27] did not show any statistically significant difference in union rate between 1,2-ICSRA and 2,3-ICSRA.

### Aim

The aim of this study was to compare the patient outcome scores between 1,2-ICSRA and 2,3-ICSRA PVBG in the treatment of scaphoid fracture non-union. Due to their theoretical advantages over 1,2-ICSRA, the hypothesis is that 2,3-ICSRA should give superior patient perceived outcome when compared with 1,2-ICSRA.

## Methods

### Study design

This was a retrospective analysis of all patients treated with1,2-ICSRA or 2,3-ICSRA PVBGs for scaphoid fracture non-union. This study was conducted in a single institution between 2017 and 2021, and operated by a single senior orthopaedic surgeon. STROBE guidelines were followed.

#### Surgical techniques

All surgeries were performed under a general anaesthesia, with antibiotic prophylaxis administered within one hour of surgery. Patients were positioned supine with a pneumatic tourniquet applied to the upper arm, and hand positioned on a hand table.

##### 1,2-ICSRA-PVBG

Approach was through a curved skin incision on the radial surface on the dorsum of the wrist. Branches of the superficial branch of the radial nerve were preserved. The extensor retinaculum was incised, and the first extensor compartment tendons retracted palmarly, while the second extensor compartment tendons were retracted ulnarly. The 1,2-ICSRA was identified and dissected from the surrounding soft tissue to mobilise it. Using the radial based triangular flap technique, wrist capsulotomy was performed, and fracture site exposed. The fracture was debrided, and several drill holes made in the distal and proximal bone fragments. Cortico-cancellous bone graft based on the 1,2-ICSRA was harvested. Tourniquet was deflated and bleeding from bone graft ensured. The graft was then placed in the scaphoid non-union, reduction performed, and fixation achieved with a headless cannulated compression screw (HCCS) (TST® Istanbul, Turkey) over a guide wire.

##### 2,3-ICSRA-PVBG

Approach was through a longitudinal incision centred over the Lister’s tubercle on the dorsum of the wrist. The extensor retinaculum was incised, and second/third wrist extensor compartment space was exposed. The 2,3-ICSRA was identified and dissected from the surrounding soft tissue to mobilise it. Using the radial based triangular flap technique, wrist capsulotomy was performed, exposing the fracture site. The fracture was debrided, and several drill holes made in the distal and proximal bone fragments. Cortico-cancellous bone graft based on the 2,3-ICSRA was harvested. Tourniquet was deflated and bleeding form the bone graft ensured. The graft was then placed in the scaphoid non-union, reduction performed, and fixation achieved with a HCCS over a guide wire.

##### Postoperative management

The wrist was immobilised in a plaster of Paris cast for a total of 6 weeks. The patients were then referred to hand therapy department to start wrist range of movement exercises.

### Recruitment and data collection

Recruitment of individuals was through letters sent by post containing information about this study. Those who agreed to take part in this study, filled in two questionnaires, and sent it back via a self-addressed envelope which was provided.

### Participants

All individuals who were 18 years or older at the time of recruitment and who were treated using 1,2-ICSRA-PVBG, or 2,3-ICSRA-PVBG for scaphoid non-union during the years 2017 and 2021 by only one specific senior orthopaedic surgeon in a single institution were eligible to take part in this study. Those who were under the age of 18 years at the time of recruitment or operated by other orthopaedic surgeon or different institution were excluded from this study. The time from injury to surgery was more than 4 months with CT-scan showing no signs of any bony healing.

### Data source

The data source used in this study was from two questionnaires sent to the participants by post. These were the Modified mayo wrist (MMW) and the patient rated wrist evaluation (PRWE) scores. Both questionnaires were used to gauge the patient perceived hand and wrist functional outcome after surgery. The PRWE is a 15-item questionnaire used to gauge wrist pain and disability during normal daily activities. Each item is rated by the patient from 0 (no pain or difficulty) to 10 (severe pain or inability to carry out the task). This questionnaire contains two subscales. The first 5 items make up the pain subscale, while the remaining 10 items make up the functional subscale. In the PRWE the higher the score the worse the functional outcome. The MMW score measures the patient work status, wrist range of motion, grip strength, work status, and pain. In the MMW score the higher the score the better the functional outcome. The final score is then graded into excellent (score of 90–100), good (score of 80–89), fair (score of 65–79), and poor (score of less than 65).

### Statistical methods

Analysis of the data was performed using IBM® SPSS® version 27. Percentages, frequencies, means, and ranges were used to analyse the demographical data. Mann-Whitney U test was used to test for statistically significant difference in the scores between the two PVBGs. A statistically significant difference was accepted at p-values of less than 0.05.

## Results

### Participants

Questionnaires were sent to the 32 individuals who underwent 1,2- or 2,3-ICSRA-PVBG between 2017 and 2021in our institution by a single surgeon. The response rate for the patient-rated questionnaires was 59% (n = 19), which was 67% (n = 8) in the 1,2-ICSRA group, and 55% (n = 11) in the 2,3-ICSRA group.

### Descriptive data

All patients were males with a mean age of 25.5 years (range 18–59). The mean age of the individuals in the 2,3-ICSRA was 7 years less than the mean age of those in the 1,2-ICSRA group (22.5 years versus 29.6 years). Four patients had other procedures done during the same surgery. Two patients in the 1,2-ICSRA group had concomitant radial styloidectomy, while the other two in the 2,3-ICSRA had concomitant scapho-lunate ligament reconstruction (see Table 1). From all the questionnaires that were returned, there was no missing data.

**Table 1:** Demographic data

	1,2-ICSRA	2,3-ICSRA	Total
**No. of patients recruited**	12	20	32
**No. of patients who accepted to take part in study**	8	11	19
**Percentage (%)**	42%	58%	100
**Males**	8	11	19
**Females**	0	0	0
**Mean age (years)**	29.6	22.5	25.5
**Age range (years)**	18–59	18–28	18–59
**Other procedures**	2	2	4

### Outcome data

In this study there was only one outcome event. This was a questionnaire sent by post in 2022 to assess the patient perceived functional outcome following surgery.

### Main results and other analyses

In the MMW score 87.5% (n = 7) of those in the 1,2-ICSRA group attained an excellent score, 12.5% (n = 1) attained a good score, and none attained a fair or poor score. In the 2,3-ICSRA group, however, only 9% (n = 1) achieved an excellent score, 36.4% (n = 4) achieved a good score, another 36.4% (n = 4) achieved a fair score, while 18.2% (n = 2) had a poor score.

The Mann-Whitney U test was used to test for any statistically significant difference in scores between the two PVBG groups. Using p-value of 0.05, the null hypothesis was rejected when the U-value of less than 19. When testing the MMW score between the two PVBG, a statistically significant difference was found (U-value of 10.5) with the 1,2-ICSRA group having better scores. The sub-scores of the MMW score were also tested for statistically significant difference. There was no statistically significant difference in the pain, function, and grip sub-scores (U-values of 28, 40 and 20 respectively). However, there was a statistically significant difference in the range of motion sub-score (U-value of 15.5). Unlike the MMW score, there was no statistically significant difference in the PRWE scores between 1,2- and 2,3-ICSRA groups (U-value of 24). The pain, function, and activities sub-scores of the PRWE also did not result in a statistically significant difference between the two PVBG (U-values of 28, 23 and 22.5 respectively).

## Discussion

### Key results

There was no statistically significant difference in the PRWE score or its pain and function sub-scores between the two PVBGs. However, a statistically significant difference in the MMW score between the two PVBGs was found, with all patients in the 1,2-ICSRA group attaining a good to excellent MMW score, and only 45.4% achieving a good to excellent MMW score in the 2.3-ICSRA group. The MMW sub-score that was statistically significant was the range of motion, while the pain, function, and grip sub-scores were not statistically significant.

### Limitations

A main limitation of this study is the small sample size. This is due to the relatively low numbers of scaphoid non-unions that get referred to our institution, and due to the 59% response rate to the mailed questionnaire. Despite a 59% response rate for mailed questionnaires is considered as an excellent response rate, however, it means that we failed to gather data on the remaining 41% of patients. This could have been improved with a different data collection method, such as questionnaires being filled in during clinic follow up. However, this was not possible as only data collection by mailed questionnaire was approved by our institutional data protection unit.

This study also, did not gather information about patient co-morbidities, such as smoking which could adversely affect bone healing. Surgery was performed after 4 months from injury, however, this study did not look into the chronicity of the injury, which could also have an impact on outcome between the two groups. There was also unequal follow up of patients, as the questionnaire was mailed in 2022, and pre-operative values of the PRWE and MMW scores were not available. Ideally the post-operative questionnaire results would be compared with the pre-operative questionnaire results to assess for improvement between the two PVBGs.

This study focused on the patient perceived outcome score between the two PVBGs. It would have been improved by including data on union rate and time to union. This information would have shed a light on whether outcome scores are related to union.

### Strengths

The strength of this study is that the surgeries were performed by only one surgeon and performed in the same hospital. This avoided different outcomes due to variations in the surgical techniques if the surgeries were performed by multiple surgeons. On the other hand including multiple surgeons apart from having higher number of patients, it would have better emulated the real situation, and a comparison between performance by different surgeons and patient outcome could be made.

### Interpretation

Tan and Tu [11] were the very few who reported their experience with using 2,3-ICSRA in scaphoid fracture non-union. They reported a series of 52 patients with an average age of 38 years. Fixation of the scaphoid and graft was with a 3.0 mm cannulated compression screw. The graft was then secured by inserting one or two 1.3/1.5 mm mini screws in the distal and/or proximal pole of the scaphoid. This was done as the authors believed that the mini screws enabled early rehabilitation by preventing bone graft from displacing. Union was attained in 48 patients (92.3%) at 14.5 weeks. The MMW score was excellent in 32 patients, good in 13 patients, fair in 3 patients, and poor in 4 patients. This means that 86% of patients attained good to excellent MMW score. This contrasts the findings of our study, were good to excellent scores were only achieved in 45.5% of patients who were treated with 2,3-ICSRA.

The discrepancy in outcome between the study of Tan and Tu [11] and this study, could have arisen from the difference in fixation methods. In this study the scaphoid and graft were fixed with only one cannulated compression screw, while in the study by Tan and Tu [11] this was supplemented by one or two mini screws to secure the graft in place. This additional fixation might have reduced graft motion and avoided twisting of the 2,3-ICSRA vascular pedicle, leading to better outcome. The MMW score is a clinician-rated score, however, in this study it was modified into a patient rated score, therefore, the range of motion and grip strength were not accurately measured using a goniometer and Jamar hand dynamometer, but as perceived by the patient. This could have been the main reason for the discrepancy in MMW score between this study and the study of Tan and Tu. [11].

## Conclusion

Despite the 2,3-ICSRA having a longer arc of rotation, relatively easier technique, and longer nutrient arteries, in this questionnaire-based study when compared with the 1,2-ICSRA as a PVBG for the treatment of scaphoid fracture non-union, it did not outperform the 1,2-ICSRA. In fact, in the MMW score, the 1,2-ICSRA outperformed the 2,3-ICSRA, mainly due to better range of motion. However, these results must be interpreted with caution considering this study was retrospective with a low sample size.

## Data Availability

Anonymized data sets used in this study are available from corresponding author on reasonable request.
